# Evaluation of Optical Low Coherence Reflectometry Parameters in Patients with Exfoliation Syndrome

**DOI:** 10.1155/2015/658091

**Published:** 2015-04-28

**Authors:** Emrullah Beyazyıldız, Özlem Beyazyıldız, Süleyman Günaydın, Emrah Kan, Mert Şimşek, Pelin Yılmazbaş, Faruk Öztürk

**Affiliations:** ^1^Department of Ophthalmology, Samsun Training and Research Hospital, Samsun, Turkey; ^2^Department of Ophthalmology, Ulucanlar Research and Training Hospital, Ankara, Turkey; ^3^Department of Ophthalmology, Ataturk Research and Training Hospital, Ankara, Turkey

## Abstract

*Purpose*. To evaluate optical low coherence reflectometry (OLCR) parameters in patients with exfoliation syndrome (EXS) undergoing cataract surgery. *Methods*. Forty-seven eyes of 47 patients with EXS (Group 1), and 55 eyes of 55 healthy subjects (Group 2) were included in the study. Anterior chamber depth (ACD), lens thickness (LT), axial length (AL), central corneal thickness (CCT), horizontal corneal length (HCL), and pupil diameter (PD) parameters were measured by OLCR (Lenstar LS 900, Haag-Streit) and compared between groups. Shapiro-Wilk test and Mann Whitney *U* tests were used for statistical analyses. *Results*. The mean ACD, HCL, and PD values were significantly lower in EXS group than in healthy subjects (*P* = 0.01, *P* = 0.04, and *P* < 0.001, resp.). The mean LT was significantly higher in EXS group than in healthy subjects (*P* = 0.007). There was no significant difference between groups in means of AXL and CCT. *Conclusions*. According to OLCR measures, eyes with EXS have shallower ACD, smaller PD, thicker LT, shorter HCL, and no significantly different CCT levels.

## 1. Introduction

Exfoliation syndrome (EXS) is a systemic disease associated with the accumulation of microfibrillar materials on systemic and ocular tissues [1]. EXS may accumulate diffusely in anterior segment of the eye and thus may be a risk factor for complications in cataract surgery. The most important risk factor for vitreous loss during cataract surgery is small pupil size, zonular weakness, and brittle lens capsule [2, 3]. Deposited microfibrillary material in anterior segment of the eye leads to weakening of zonular support and laxity of the lens and consequent shifting of the lens anteriorly [4].

Shallower anterior chamber has been shown as indicator for zonular instability in eyes with EXS [4]. Therefore, evaluation of anterior segment parameters is essential in refractive surgeries and accurate measurements are needed to obtain satisfactory postoperative results. New optical low coherence reflectometry (OLCR) Lenstar LS 900 (Haag-Streit) is able to measure anterior chamber depth (ACD), lens thickness (LT), axial length (AL), central corneal thickness (CCT), horizontal corneal length (HCL), and pupil diameter (PD). The aim of this study was to evaluate OLCR parameters of eyes with EXS eyes undergoing cataract surgery for accurate measurements for obtaining good postoperative results.

## 2. Materials and Methods

One hundred and two eyes with cataract undergoing cataract surgery were included in this cross-sectional study. All patients were Caucasians. Eyes with EXS (*n* = 45) were classified as group 1 and eyes with only cataract (*n* = 57) were classified as group 2. All of the patients were evaluated with OLCR (Lenstar LS 900, Haag-Streit), preoperatively by the same physician. Best corrected visual acuity, intraocular pressure (IOP), anterior and posterior segment findings, and OLCR measures (ACD, LT, AL, CCT, HCL, and PD) of patients were noted and compared between groups. Measurements were performed with a dilated pupil. Study was approved by the local ethical committee and informed consent was obtained from all of the subjects.

Inclusion criteria for EXS group were occurrence of exfoliative material on the anterior capsule or at the pupillary border and cataract. All patients had healthy, normal optic discs, defined as having a linear cup-disk ratio < 0.5, healthy neuroretinal rim, and no glaucomatous findings such as peripapillary hemorrhages, thinning, and localized loss of neuroretinal rim. Exclusion criteria were presence of glaucoma, previous history of ocular trauma and ocular surgery, and ocular disease which may affect anterior segment parameters.

### 2.1. Statistical Analysis

Independent samples *t*-test and the Mann-Whitney *U* tests were used for comparing variables between two groups. The Shapiro-Wilk test and Mann-Whitney *U* tests were used for statistical analysis of difference in OLCR parameters of groups. A two-tailed probability of 0.05 was considered as statistically significant.

## 3. Results

There were 47 eyes in subjects with EXS (group 1) and 55 healthy subjects in control group (group 2). Mean age in group 1 was 73.0 ± 8.08 and 71.2 ± 9.58 in group 2 with no significant difference (*P* = 0.43). Female/male ratio was similar in both groups (*P* = 0.61). There was no significant difference between groups regarding IOP and BCVA (*P* = 0.20 and *P* = 0.43, resp.) Table 1.

According to OLCR measures, mean AXL in group 1 was 23.2 ± 0.93 and 23.3 ± 0.97 in group 2 with no significant difference (*P* = 0.68). The mean CCT in group 1 was 527 ± 30.7 and 524 ± 0.32 in group 2 with no significant difference (*P* = 0.68). The mean ACD in group 1 was 3.19 ± 0.40 and 3.40 ± 0.46 in group 2. Eyes with EXS had significantly shallower ACD (*P* = 0.01). The mean LT in group 1 was 4.40 ± 0.37 and 4.18 ± 0.44 in group 2. Eyes with EXS had significantly thicker lens (*P* = 0.007). The mean HCL was 11.7 ± 0.50 in group 1 and 11.9 ± 0.46 in group 2 (*P* = 0.04). The mean PD in group 1 was 4.86 ± 1.02 and 5.84 ± 1.27 in group 2. Eyes with EXS had significantly smaller PD (*P* < 0.001) Table 2.

Posterior capsule rupture was observed in 8 patients during cataract surgery (3 in group 1 and 5 in group 2). There was no significant difference between groups (*P* = 0.74). Two cases of dropping of lens into vitreous were observed in group 1 and no case of lens drop was observed in group 2 Table 3.

## 4. Discussion

In this study we have evaluated OLCR findings of eyes with EXS undergoing cataract surgery. As EXS could be seen in up to 25.9% incidence in people aged over 60 [5], surgeons should be aware of changes of anterior segment parameters in EXS patients because of increased complication rates due to accumulation of exfoliative material in anterior segment, lens capsule, and zonules [6]. In our study, although not significantly, eyes with EXS had higher IOP compared to control subjects. Intraocular pressure in EXS should be considered with corneal biomechanical properties as these properties have great influence on IOP [7]. There are reports in the literature showing thinner CCT in eyes with EXS [8, 9] whereas Puska et al. reported that eyes with EXS have higher CCT [10]. So there are many conflicting reports in the literature about CCT in eyes with EXS. In our study there were no significant different measures of CCT observed in eyes with EXS syndrome from healthy subjects. Conflicting reports in the literature may be due to variable study groups and multiple factors effecting CCT values of the eyes.

Zheng et al. have evaluated eyes with EXS and compared them with controls. They have found that eyes with EXS have shallower anterior chamber than healthy eyes in concordance with our study [11]. There are many studies reporting increased incidence of complications during cataract surgeries in eyes with EXS [12–15]. Shallower anterior chamber may be a risk factor for complications during cataract surgeries of eyes with EXS. In a different study association between anterior chamber depth and complications during cataract surgery in eyes with EXS has been evaluated [4]. According to this study results increased complication rates during cataract surgery were observed in eyes with shallower anterior chamber. Shallower ACD in eyes with EXS might be associated with zonular laxity and careful preoperative evaluation of ACD in these eyes may be helpful for successful outcome of cataract surgery. Smaller pupil size in these eyes may also be related with increased rates of complications. In our study EXS eyes had significantly shallower anterior chamber and smaller pupil diameter. Degenerative changes in both stromal and muscular layers of the iris have been shown in iris of the EXS eyes [16]. In a different study mechanical restriction of pupil has been attributed to the adherent material between pigment epithelium of the iris and anterior lens capsule [17, 18]. Accommodation amplitude of the eyes with EXS has also been found to be decreased compared to healthy subjects [19].

Decreased accommodation amplitude leads to asthenopic complaints and blurred vision at near in these subjects [20]. Accommodation amplitude has been shown to be related with a pupil diameter and smaller pupil size leads to an increase in the measured accommodation amplitude [21]. Yavas et al. did not find any correlation between accommodation and pupil diameter in EXS eyes [19].

Although Yavas et al. found that LT was not significantly different in EXS eyes from healthy subjects, in our study EXS eyes have significantly thicker lenses [19]. There are conflicting reports in the literature about LT in EXS eyes. According to the results of Reykjavik Eye Study of 493 EXS eyes, EXS eyes have thicker lens compared to healthy subjects, but after adjustment for age, there was no significantly different lens thickness in EXS eyes compared to healthy subjects [22]. In our study EXS group had a higher mean age level, but there was no significant difference between groups regarding age. So we did not make any adjustments in statistical analysis for age. Bosnar et al. have analysed 47 eyes of EXS and in concordance with our study have found thicker LT in EXS eyes [17]. In our study, There were 5 complications in either group and no significant difference observed between groups related with complications. This result may be due to small number of study groups. Small PD and phacodonesis were the most important risk factors for complications during cataract surgery in eyes with EXS [23].

Careful evaluation of OLCR measures should be undertaken in patients with EXS as shallower ACD and small PD may be an indication of zonular weakness. Shallower ACD from 2.5 mm in eyes with EXS have been associated with an increased risk of complication during cataract surgery [24]. It was hypothesized that phacodonesis and anterior lens displacement due to zonular weakening may be a reason for shallower ACD in EXS [17]. OLCR measures could be a useful predictor of intraoperative complications. Precautions could be undertaken during preoperative and intraoperative period, such as capsular tension ring placements in patients with zonular instability suspects, iris hooks, or pupillary ring placements to ensure adequate dilation in patients with small pupils with the help of OLCR evaluation.

To achieve optimal postoperative results in patients undergoing cataract surgery, accurate measurements of ocular parameters are essential in modern cataract surgery. Measurement with OLCR seems to be a safer and practical tool for assessing anterior segment parameters of eyes with EXS; however its repeatability and precision for all parameters in EXS eyes should be analyzed and compared with other methods such as IOLMaster (Carl Zeiss, Jena, Germany). OLCR may fail to measure anterior segment parameters in patients with dense cataract or corneal edema and ultrasound biometry can be used in this cases. Different ethnical groups have different incidence of EXS and ocular parameters [25]. All patients in our group were with the same ethnic group; thus there is no effect of ethical differences on OLCR parameters in this study.

As a conclusion, eyes with EXS have shallower ACD, smaller PD, and thicker LT analysed by OLCR. Shallower ACD and smaller PD may be indicative of zonular weakness and increased risk for complications during cataract surgery. Optical low coherence reflectometry is a convenient device to measure anterior segment parameters of eyes with EXS during preoperative evaluation.

## Figures and Tables

**Table 1:** Demographic and clinical properties of the groups.

	Group 1 (*n* = 47)	Group 2 (*n* = 55)	*P* ^c^
Mean age	73.0 ± 8.08	71.2 ± 9.58	*P* = 0.67
BCVA^a^	0.14 ± 0.10	0.17 ± 0.13	*P* = 0.43
IOP^b^	15.0 ± 4.5	14.01 ± 3.28	*P* = 0.20
Female/male ratio	22/25	23/32	*P* = 0.61

^a^BCVA: best corrected visual acuity; ^b^IOP: intraocular pressure; ^c^statistical value.

**Table 2:** Optical low coherence reflectometry findings of patients.

	Group 1	Group 2	*P* ^g^
AXL^a^	23.2 ± 0.93	23.3 ± 0.97	*P* = 0.68
CCT^b^	527 ± 30.7	524 ± 0.32	*P* = 0.68
ACD^c^	3.19 ± 0.40	3.40 ± 0.46	*P* = 0.01
LT^d^	4.40 ± 0.37	4.18 ± 0.44	*P* = 0.007
HCL^e^	11.7 ± 0.50	11.9 ± 0.46	*P* = 0.04
PD^f^	4.86 ± 1.02	5.84 ± 1.27	*P* < 0.001

^a^AXL: axial length; ^b^CCT: central corneal thickness; ^c^ACD: anterior chamber depth; ^d^LT: lens thickness; ^e^HCL: horizontal corneal length; ^f^PD: pupil diameter; ^g^statistical value.

**Table 3:** Incidence of complications during cataract surgery.

	Group 1	Group 2	*P* ^b^
PCR^a^	3 (6.4%)	5 (9.1%)	*P* = 0.74
Lens drop	2 (4.3%)	—	—
Iris damage/zonular dialysis	—	—	—

^a^PCR: posterior capsule rupture; ^b^statistical value.
